# Is it time to consider the expression of specific-pituitary hormone genes when typifying pituitary tumours?

**DOI:** 10.1371/journal.pone.0198877

**Published:** 2018-07-06

**Authors:** Araceli García-Martínez, Johana Sottile, Carmen Fajardo, Pedro Riesgo, Rosa Cámara, Juan Antonio Simal, Cristina Lamas, Hernán Sandoval, Ignacio Aranda, Antonio Picó

**Affiliations:** 1 Research Laboratory, Hospital General Universitario de Alicante-Institute for Health and Biomedical Research (ISABIAL), Alicante, Spain; 2 Endocrinology Department, Hospital de La Ribera, Alzira, Valencia, Spain; 3 Neurosurgery Department, Hospital de La Ribera, Alzira, Valencia, Spain; 4 Endocrinology Department, Hospital Universitario y Politécnico La Fe, Valencia, Spain; 5 Neurosurgery Department, Hospital Universitario y Politécnico La Fe, Valencia, Spain; 6 Endocrinology Department, Complejo Hospitalario Universitario de Albacete, Albacete, Spain; 7 Neurosurgery Department, Complejo Hospitalario Universitario de Albacete, Albacete, Spain; 8 Pathological Department, Hospital General Universitario de Alicante, Alicante, Spain; 9 Endocrinology Department, Hospital General Universitario de Alicante-Institute for Health and Biomedical Research (ISABIAL), Miguel Hernández University, CIBERER, Alicante, Spain; University of Cordoba, SPAIN

## Abstract

The aim of the present study is to check whether we can replicate, in an independent series, previous results showing that the molecular study of pituitary-specific gene expression complements the inmunohistochemical identification of pituitary neuroendocrine tumours. We selected 112 patients (51 (46.4%) women; mean age 51.4±16 years; 102 macroadenomas (91.9%), 9 microadenomas (8.1%)) with complete clinical, radiological, immunohistochemical and molecular data from our data set of pituitary neuroendocrine tumours. Patients were different from those previously studied. We measured the expression of the pituitary-specific hormone genes and type 1 corticotrophin-releasing hormone and arginine vasopressin 1b receptors, by quantitative real-time polymerase chain reaction using TaqMan probes. Afterwards, we identified the different pituitary neuroendocrine tumour subtypes following the 2017 World Health Organization classification of pituitary tumours, calculating the concordance between their molecular and immuhistochemical identification. The concordance between molecular and immunohistochemical identification of functioning pituitary neuroendocrine tumours with the clinical diagnosis was globally similar to the previous series, where the SYBR Green technique was used instead of TaqMan probes. Our results also corroborated the poor correlation between molecular and immunohistochemical detection of the silent pituitary neuroendocrine tumour variants. This discrepancy was more remarkable in lactotroph, null-cell and plurihormonal pituitary neuroendocrine tumours. In conclusion, this study validates the results previously published by our group, highlighting a complementary role for the molecular study of the pituitary-specific hormone genes in the typification of pituitary neuroendocrine tumours subtypes.

## Introduction

In 2017, the World Health Organization (WHO) classification of tumours of the pituitary gland superseded its previous 2004 version[1]. In fact, in the past several decades, advances in identification techniques for anterior pituitary neoplasms have prompted several changes in the classification of these tumours. The 2004 WHO classification was mainly based on the immunohistochemical staining of the anterior pituitary hormones in the tumour. Despite the great progress made in immunohistochemistry (IHC), this technique continues to rely on the skills of the observer, the antibodies used, and on the protein expression of the tumour cells. Therefore, it insufficiently identified null cell tumours (NC) and plurihormonal (PH) tumours. Furthermore, it insufficiently predicted the clinical behaviour of atypical neoplasm subtypes, giving rise to proposals for a new terminology of the anterior pituitary neoplasms: pituitary neuroendocrine tumour (PitNET)[2].

To overcome some of these limitations, the new 2017 WHO classification recommends IHC determination of the pituitary-specific transcription factors[1], followed by subclassification of the different PitNET subtypes. Pituitary-specific transcription factors are useful markers for identifying the cytogenesis of pituitary tumours[3]. Tpit specifically regulates the expression of *POMC* in corticotroph tumours (CT). Neurod1 contributes to the differentiation of adrenocorticotropic hormone (ACTH)-secreting tumours and gonadotroph tumours (GT)[4]^,^[5]^,^[6]. Steroidogenic factor 1 (SF-1) is expressed in GT, and Pit1 is expressed in tumours derived from the production lines of growth hormone (GH) (somatotroph tumours, ST), prolactin (PRL) (lactotroph tumours, LT) and thyroid-stimulating hormone (TSH) (thyrotroph tumours, TT)[7]^,^[8]. Pit1 acts synergistically with the oestrogen receptor and the GATA-2 binding protein to induce the expression of prolactin) and TSH, respectively[9].

Immunohistochemical determination of the specific transcription factors of the anterior pituitary hormones may improve identification of NC and PH. However, it will continue to present the limitations of IHC as a semiquantitative and observer-dependent technique. In addition, many of the antibodies against transcription factors are not commercialised, constraining the possibility to properly classify PitNET to a few reference centres[10].

Currently, the molecular and genetic characteristics of tumours are considered a diagnostic tool that complements pathological diagnosis[11]. Applied to the pituitary gland, the study of pituitary-specific gene expression would allow identifying the source line of the NC. In addition, it could contribute to the better typification of the PH tumours. In fact, Sánchez-Tejada *et al*.[12] have recently published a molecular identification of PitNET subtypes that reduces the number of NC and PH with respect to their IHC identification.

The aim of the present study is to check whether the results obtained by Sanchez-Tejada *et al*. can be replicated in an independent series, carried out by a different researcher and with certain methodological changes. More broadly, we aim to contribute to improving the diagnosis of these tumours by incorporating molecular biology techniques that may be applicable in clinical practice.

## Material and methods

### Patients and tumours

From our collection of PitNET, we studied 118 patients (51 (46.4%) women; mean age 51.4±16 years; 102 macroadenomas (91.9%), 9 microadenomas (8.1%)) with complete clinical, immunohistochemical and molecular data. The rest of the patients of the collection had already been studied[12]. We identified the tumours–different from those studied by Sanchez-Tejada et al[12]–from four reference hospitals for pituitary surgery (Hospital General Universitario de Alicante, Hospital Universitario de la Ribera, Hospital Universitario y Politécnico La Fe, and Complejo Hospitalario Universitario de Albacete). We collected anonymised clinical, pathological and radiological data (age, gender, clinical and IHC subtype, tumour size) for each sample from the Spanish Molecular Registry of PitNET (REMAH) database, which is part of a Spanish multicentre project[13]. Six patients were excluded because the tumours were contaminated with normal pituitary tissue. We defined tumour contamination with normal pituitary tissue according to the pathologist’s judgment and when the expression of specific-pituitary genes was complete for all hormones without predominance of any of them[14]. Therefore, the study was performed in 112 PitNET.

The study complies with the Declaration of Helsinki and other applicable laws and has received the approval of the local ethics committee (CEIC General University Hospital of Alicante). None of the donors came from a vulnerable population, and all donors or relatives freely provided written informed consent.

### IHC and molecular studies

The IHC studies took place in the pathology departments of the four participating reference hospitals. Formalin-fixed paraffin-embedded (FFPE) tissue from tissue microarrays was used with standard automated techniques in the Autostainer Link48 (Dako-Agilent) with the Envision (Dako) high-sensitivity visualisationg system. S1 Table details the information on the antibodies used.

All molecular studies were centralised in the laboratory of the Research Institute of the General University Hospital of Alicante. A single investigator analysed the expression of the GH, follicle stimulating hormone (FSH), luteinising hormone (LH), PRL, TSH and *AVPR1*, *CRHR1* and *POMC* genes in all tumours.

#### RNA extraction and cDNA synthesis

All samples were preserved immediately after surgery in RNAlater solution at 4°C for 24 hours and then stored at −20°C. The biological samples were disintegrated in the TissueLyser (Qiagen, Hilden, Germany). We used the AllPrep DNA-RNA-Protein kit (Qiagen) for manual RNA extraction and measured the concentration and purity of the RNA in the Nanodrop spectrophotometer (Thermo Scientific, Waltham, MA, USA).

For each retrotranscription reaction, we used 2 μg of RNA in a total volume of 20 μL, employing the High-Capacity cDNA Reverse Transcription kit (Applied Biosystems).

#### Quantitative real-time polymerase chain reaction (qRT-PCR)

We performed qRT-PCR following the manufacturer's instructions in the 7500 Fast Real-Time PCR system (Life Technologies, CA, USA) and used TaqMan Fast Advanced PCR Master Mix and assays based on hydrolysis probes (TaqMan Gene Expression Assays, Life Technologies, CA, USA). We selected the following assays: GH (1573905 A6), *FSHB* (Hs00174919_m1), *LHB* (Hs00751207_s1), *TSHB* (Hs02759015_s1), PRL (Hs00168730_m1), *POMC* (Hs01596743_m1), *AVPR1B* (Hs00949767_m1) and *CRHR1* (Hs00366363_m1). Reference genes used were: *PGK1* (Hs00943178_g1), *TBP* (Hs00427620_m1) and *MRPL19* (Hs00608519_m1). As a positive control, we used total RNA from normal adult brain tissue (Bionova). A pool of RNA from nine normal pituitary samples obtained from autopsies served as a calibrator. All samples were analysed in duplicate. The relative differences in gene expression were expressed as fold change and were obtained with the 2^-ΔΔCt^ method (SDS software, Applied Biosystems).

### Clinical, molecular and IHC classification of PitNET and concordance between them

The attending clinicians classified the tumours as functioning PitNET (FPitNET) or non-functioning PitNET (NFPitNET), depending on whether the patients presented with a recognisable clinical and biochemical endocrine syndrome, namely Cushing syndrome, acromegaly, hypogonadism-amenorrhea or clinical hyperthyroidism.

We relied on the dominant gene expression in the qRT-PCR or on the dominant protein expression in the IHC of the anterior pituitary hormones to identify the different subtypes of the PitNET. Namely, GH in somatotroph (ST), PRL in lactotroph (LT), TSH in tirotroph (TT), ACTH in corticotroph (CT) and FSH or LH in gonadotroph (GT) PitNET. Afterwards, we followed the new 2017 WHO classification of pituitary tumours to group the tumours according to their molecular and IHC characteristics.

When the ST showed dominant co-expression of the PRL gene or immunoreactivity higher than 5% for PRL, we considered the tumour as a mixed ST (ST Mixed) PitNET. When the LT showed co-expression of the GH gene or immunoreactivity higher than 5% for GH (focal and variable), we considered the tumour an LT stem cell (LT STEM) PitNET.

When the tumour showed dominant gene expression or immunoreactivity for more than one pituitary hormone coming from the same pituitary transcription factor, i.e. GH/PRL/TSH, we considered the tumour as plurihormonal PIT1 (PH-PIT1) PitNET.

When the tumour showed dominant gene expression or immunoreactivity for more than one pituitary hormone coming from different pituitary transcription factors, i.e. ACTH/GH, ACTH/PRL, FSH/PRL, we considered the tumour as an unusual plurihormonal (U-PH) PitNET.

When the tumour did not show dominant gene expression or did not express immunoreactivity for any of the pituitary hormones, we considered the tumour as an NC PitNET.

Finally, we typified the molecular or IHC forms of PitNET that did not express any endocrine syndrome as silent variants (i.e. S-ST, SCT etc.) of the corresponding functioning ones (i.e. FST, FCT, etc.) (S2 Table).

Once we identified the functioning and non-functioning (silent) variants of the different subtypes of PitNET, we calculated their prevalence in the sample according to their molecular or IHC typification.

Once we had classified all the tumours of the collection according to 2017 WHO classification, we studied the concordance between the three methods of identification using the same strategy as Sanchez-Tejada et al[12]. In short, we calculated the concordance between the clinical identification of the tumours with the IHC and molecular ones in the case of functioning tumours. The functioning PH tumours (PH-PIT1 or U-PH) were considered as their respective functioning subtypes depending on the dominant gene expression or proteins. Afterwards, we only calculated the concordance between the IHC identification with the molecular one in the case of non-functioning tumours.

As previous medical treatment with somatostatin analogues or cabergoline could down-regulate the expression of the GH or PRL genes[15]^,^[16]^,^[17]^,^[18]^,^ [19]^,^[20]^,^[21], we divided the cohort of FST in two sub-cohorts according to whether they had or had not been treated with lanreotide/octreotide before surgery. However, all except one functioning lactotropinoma (FLT) of the series had been treated prior to surgery with cabergoline. Consequently, we assessed the effect of the cabergoline treatment on the expression of the PRL gene by means of a linear regression between the cumulative doses of cabergoline (mg per week × number of weeks) administered prior to surgery and the amount of fold change of the PRL gene.

### Statistical analysis

Qualitative variables, including PitNET subtypes, were expressed as absolute and relative frequencies. Participant age was expressed as mean ± standard deviation (SD). Molecular variables showed a non-normal distribution (Kolmogorov-Smirnov test), so we chose percentiles as the measure of distributions and reported them as median plus interquartile range (IQR). We calculated the Cohen’s kappa coefficient to measure concordance between the clinical, IHC and molecular identification of the different subtypes of PitNET (κ = 1 representing complete concordance and κ ≤ 0 null concordance). To determine the relationship between the administered doses of cabergoline and the expression of the PRL gene in FLT, we used Spearman’s correlation. In order to determine differences in the expression of *GH* gene in functioning ST PitNET depending on the treatment with somatostatin analogues, we used the Mann Whitney test. We considered *P* values of less than 0.05 to be statistically significant. Statistical analysis was performed with SPSS 24.0 software (IBM Software).

## Results

### Clinical identification of the PitNET of the series

The series studied in the present work was composed of 112 tumours, 48 (42.8%) of which were clinically functioning. The demographic and clinical characteristics of the patients are shown in S3 Table.

### IHC and molecular identification of PitNET

Following the 2017 WHO system, we classified the different subtypes of PitNET according to the dominant pituitary-specific hormone gene or protein expression in the IHC and molecular studies (Table 1). In S4 Table, we show the expression of the dominant gene with respect the other pituitary hormone gene in the different subtypes of PitNET.

**Table 1:** PitNET subtypes identified by the immunohistochemical and molecular studies in the present series.

PitNET subtypes	Molecular n (%)	IHC n (%)
ST	12 (10.7)	14 (12.5)
ST Mixed	7 (6.3)	12 (10.7)
CT	13 (11.6)	9 (8.0)
LT	10 (8.9)	7 (6.3)
LT Stem	1 (0.9)	2 (1.8)
TT	9 (8.0)	2 (1.8)
GT	31 (27.7)	26 (23.2)
NC	14 (12.5)	19 (17.0)
U-PH	11 (9.8)	19 (17.0)
PH-PIT1	4 (3.6)	2 (1.8)
**TOTAL**	**112 (100.0)**	**112 (100.0)**

PitNET: pituitary neuroendocrine tumour; IHC: immunohistochemistry; GT: gonadotropinoma; NC: null cell tumour; ST: somatotropinoma; ST Mixed: somatotropinoma mixed; ST: somatotropinoma; CT: corticotropinoma; LT: lactotropinoma; LT Stem: lactotropinoma stem cell; TT: thyrotropinoma; U-PH: unusual plurihormonal tumour; PH-PIT1: plurihormonal PIT1 tumour.

### Classification and prevalence of the different PitNET subtypes according to their clinical, molecular, and IHC identification

Once we identified the different subtypes of PitNET by IHC and molecular study, the clinical and biochemical information allowed us to define the functioning and silent variants of the tumours. Consequently, we show the classification and prevalence of the different PitNET subtypes according to their clinical, molecular and IHC identification (Table 2).

**Table 2:** Classification and prevalence of the different PitNET subtypes according to their clinical (functioning and silent), molecular and IHC identification.

PitNET	Molecular n (%)	IHC n (%)
**ST**	12 (10.7)	15 (13.4)
**FST**	12 (100)	10 (66.7)
**S-ST**	0 (0)	5 (33.3)
**ST MIXED**	7 (6.3)	11 (9.8)
**FST MIXED**	7 (100)	11 (100)
**S-ST MIXED**	0 (0)	0 (0)
**CT**	13 (11.6)	9 (8)
**FCT**	7 (53.8)	3 (33.3)
**SCT**	6 (46.2)	6 (66.7)
**LT**	10 (8.9)	8 (6.3)
**FLT**	4 (40)	5 (62.5)
**SLT**	6 (60)	3 (37.5)
**FLT STEM**	1 (0.9)	1 (0.9)
**TT**	9 (8)	2 (1.8)
**FTT**	3 (33.3)	0 (0)
**STT**	6 (66.7)	2 (100)
**GT**	31 (27.7)	26 (23.2)
**NC**	14 (12.5)	19 (17)
**PH**	15 (13.4)	21 (18.7)
**PH PIT1**	4 (26.7)	2 (9.5)
**FPH PIT1**	3 (75)	2 (100)
**SPH PIT1**	1 (25)	0 (0)
**U-PH**	11 (73.3)	19 (90.5)
**U-FPH**	3 (27.3)	11 (57.9)
**U-SPH**	8 (72.7)	8 (42.1)

PitNET: pituitary neuroendocrine tumour; IHC: immunohistochemistry; F: functioning; S: silent; GT: gonadotropinoma; NC: null cell tumour; FST: functioning somatotropinoma; FST MIXED: functioning somatotropinoma mixed; S-ST: silent somatotropinoma; FCT: functioning corticotropinoma; SCT: silent corticotropinoma; FLT: functioning lactotropinoma; FLT STEM: functioning lactotropinoma stem cell; SLT: silent lactotropinoma; FTT: functioning thyrotropinoma; STT: silent thyrotropinoma; U-FPH: unusual functioning plurihormonal tumour; U-SPH: unusual silent plurihormonal tumour; FPH PIT1: functioning plurihormonal PIT1 tumour; SPH PIT1: silent plurihormonal PIT1 tumour.

### Concordance between the different diagnostic methods for identifying PitNET subtypes

#### Concordance of clinical identification of functioning tumours with IHC and molecular identification

**Fig 1:**
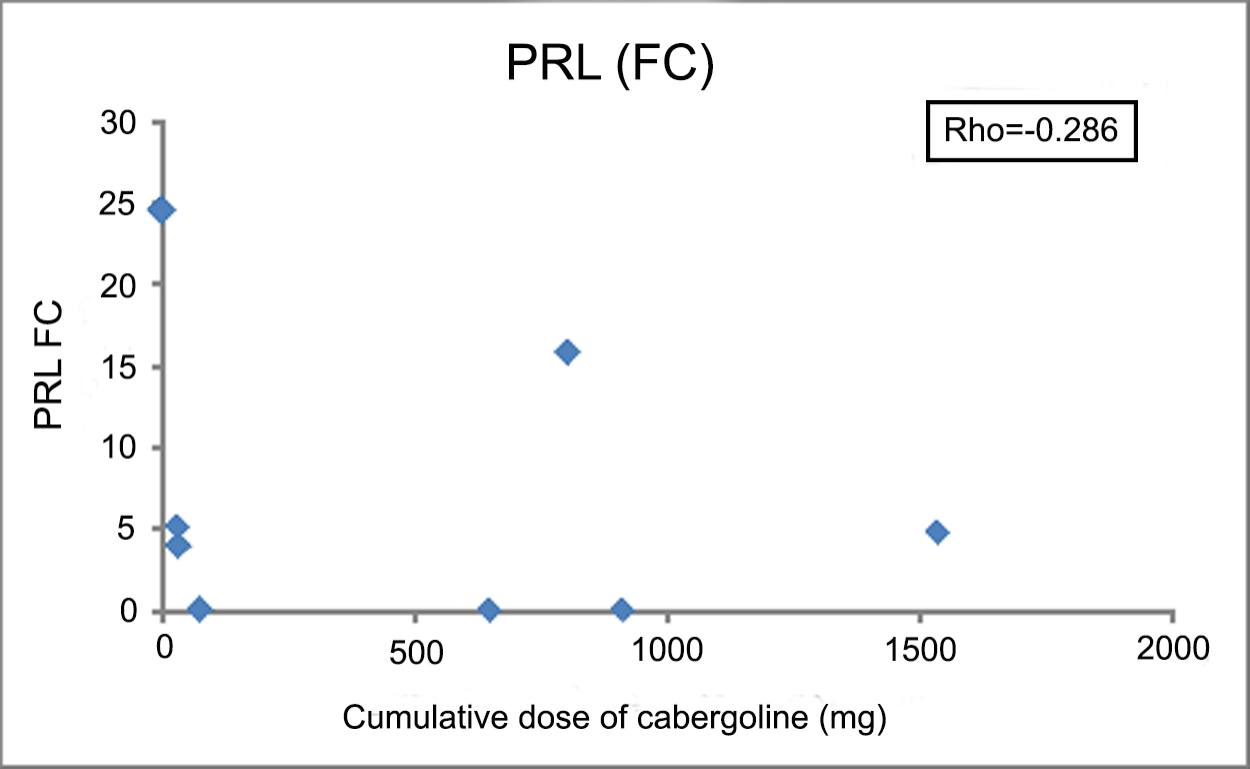
Linear regression between PRL gene expression in seven functioning lactotropinomas and the dose of cabergoline administered prior to surgery.

Table 3 shows the concordance between the IHC and molecular identification with clinical diagnosis. Overall, both IHC and molecular studies were very robust in properly identifying the functioning variants of PitNET subtypes. Only in the case of the TT subtype was molecular identification superior. In the case of STs, we did not find significant differences for IHC or molecular identification based on prior treatment with somatostatin analogues. S5 Table shows the expression of the GH gene in functioning ST-PitNET according to treatment with somatostatin analogues. In the same way, treating functioning LTs with cabergoline did not affect their identification with either method (Fig 1).

**Table 3:** Concordance between IHC and molecular identification of subtypes of functioning PitNET with clinical diagnosis.

Clinical diagnosis of functioning PitNET	N	IHC (K-coeff)	N	Molecular (K-coeff)	N
**Cushing**	10	0.810	7	0.943	9
Acromegaly*	24	0.868	24	0.886	25
With treatment	9	0.840	8	0.935	8
Without treatment	15	0.809	16	0.872	17
**Prolactinoma**	8	0.848	6	0.756	5
**Thyrotropinoma**	3	0.493	1	1.000	3

Values show Cohen’s Kappa coefficient (K = 1 represents complete concordance and K≤0 represents null concordance). IHC: immunohistochemistry; K-coeff: K-coefficient

* 3 ST were excluded from the analysis because it was unknown whether patients had received or not treatment with SAA.

#### Concordance between molecular and IHC identification in functioning and silent variants of the different subtypes of pituitary tumours

Table 4 shows the concordance between molecular and IHC identification of functioning and silent PitNET subtypes. In contrast to their functioning variants, the concordance between IHC and molecular identification of the different subtypes was poor, except for the SCT subtype. The expression of the specific genes of the functioning and non-functioning (silent) variants of PitNET of the collection is shown in S6 Table.

**Table 4:** Concordance between molecular and IHC identification in functioning and silent variants of PitNET.

PitNET	IHC (N)	Molecular (N)	*K-coefficient*
FST	28	21	0.714
FCT	7	9	0.730
FLT	6	5	0.904
FTT	1	3	0.493
GT	26	30	0.304
S-ST	4	0	NCP
SCT	6	4	0.568
SLT	2	5	-0.047
STT	2	4	0.304
U-SPH	8	8	0.143
SPH PIT1	0	1	NCP
NC	16	12	0.182

Values show Cohen’s kappa coefficient (K = 1 represents complete concordance and K = 0 represents null concordance; a negative kappa value represents agreement worse than expected, or disagreement). PitNET: pituitary neuroendocrine tumour; IHC: immunohistochemical; FST: functioning somatotropinoma; FCT: functioning corticotropinoma; FLT: functioning lactotropinoma; FTT: functioning thyrotropinoma; NCP: non-calculable parameter; GT: gonadotropinoma; S-ST: silent somatotropinoma; SCT: silent corticotropinoma; SLT: silent lactotropinoma; STT: silent thyrotropinoma; U-SPH: unusual silent plurihormonal tumour; SPH PIT1: silent plurihormonal PIT1 tumour; NC: null cell tumour.

## Discussion

Our results replicate those obtained by Sanchez-Tejada *et al*.[12]. Both studies support the molecular identification of PitNET subtypes as a complementary tool to immunohistochemistry for their classification (Table 5). Although we performed PCR with TaqMan probes instead of SYBR Green Primers, the concordance with the clinical diagnosis in functioning tumours was very high and, in both cases, superior to IHC concordance. Consequently, these results, obtained in an independent series and performed by a different investigator, validate the soundness of quantifying the molecular expression of pituitary genes in the identification of the different PitNET subtypes.

**Table 5:** Concordance between molecular and IHC identification of functioning PitNET subtypes with clinical diagnosis in the present series and in the previous one.

PitNET subtype	Present series		Sánchez-Tejada series	
	*Molecular*	*IHC*	*Molecular*	*IHC*
Cushing	0.943	0.810	0.942	0.608
Acromegaly	0.842	0.880	0.943	0.698
Hypogonadism-galactorrhea	0.756	0.848	0.701	0.678
Hyperthyroidism	1.000	0.493	1.000	0.664

IHC: immunohistochemistry; PitNET: pituitary neuroendocrine tumour.

Remarkably, the concordance of the molecular analysis with the clinical diagnosis was complete or almost complete for Cushing disease and functioning thyrotropinomas, and identical in both studies. However, there was lower concordance in the case of the acromegaly and of the functioning lactotroph PitNET in both series, with minor differences between them.

Pre-surgical treatment for acromegaly with somatostatin analogues has been shown to reduce the levels of SSTR2 proteins[22] and could also downregulate the GH gene expression. That could explain the loss of concordance with the clinical diagnosis. Indeed, Ibañez-Costa et al. demonstrated decreased in vitro GH expression in somatotropinomas treated with first generation analogues of somatostatin or pasireotide[23]. However, in our study, there were no significant differences between the GH gene expression and the GH protein, immunohistochemically identified, depending on the preoperative treatment with somatostatin analogues. Consequently, the differences in the concordance of molecular identification of acromegaly between the two studies could be attributable to the different techniques used to identify the GH gene or to other, unknown reasons. In either case, the reliability for detecting a functioning somatotropinoma was higher with the molecular analysis than with the IHC one.

Whereas the effect of somatostatin on GH gene expression is unclear, dopamine suppresses PRL gene expression through the activation of intracellular signalling pathways in experimental animals[18]. The main dopamine receptor in the pituitary is the D2-type (D2R)[19]. Alternative mRNA splicing of the D2R gene produces two receptor isoforms[24]: 415- and 444-amino acid proteins. The dopamine activation of either isoform induces suppression of the PRL gene in the rat[17]. In human LT, cabergoline treatment produces a dose-dependent decrease in the prolactin regulatory element-binding protein, inhibiting the PRL promoter activity[17]. In the present study we found a clear inverse relationship between the pre-surgical treatment of LT PitNET with cabergoline and the expression of the PRL gene. Only one patient, who had received a high amount of cabergoline prior to surgery, had an elevated fold change in the PRL gene. The patient was a 38-year-old woman diagnosed in 2015 with a 5 mm microprolactinoma (PRL 126 ng/mL) and showed complete resistance to dopamine agonists (last PRL prior to surgery while taking 3.5 mg per week of cabergoline: 175 ng/mL; IHC study: 100% positivity for PRL; negativity for the rest of pituitary hormones; cytokeratin AE1/AE3 100% cytoplasmic). Therefore, the amount of the PRL gene expression in functioning LT should be interpreted in the context of previous treatment with cabergoline.

The non-functioning subtypes of the PitNET told a different story. In these tumours, the clinical endocrine syndrome did not corroborate the diagnosis of the patient. Thus, our gold standard was the IHC identification of the tumours. The concordance between molecular and IHC identification of the different subtypes of PitNET was very poor in the both series studied. Although we observed better concordance between molecular and IHC identification of the functioning subtypes than the previous series, the concordance did not improve in the non-functioning subtypes. With the exception of the silent corticotropinomas, moreover, the molecular-IHC concordance was different between the two series (Table 6). One explanation could be that the first study identified the silent variants of PitNET molecularly based on a gene expression within the IQR of the corresponding functioning variants. On the other hand, in the present study we identified the silent variants based on the dominant gene expression in absence of a recognisable endocrine syndrome. Thus, on that point there is a high variability in the IHC identification of the pituitary tumours, particularly when the IHC studies are performed in different pathology departments.

**Table 6:** Concordance between molecular and IHC identification in silent functioning PitNET subtypes in the present series and in the previous one.

PitNET subtype	Present series	Sánchez-Tejada series
SGT	0.304	0.183
SCT	0.568	0.519
STT	0.304	0.318
SLT	−0.047	NP
NC	0.182	0.259
U-SPH	0.143	NCP
SPH-PIT 1	NP	NCP

NC: null cell tumour; NCP: non-calculable parameter; S-ST: silent somatotropinoma; SCT: silent corticotropinoma; GT: gonadotroph tumours SLT: silent lactotropinoma; STT: silent thyrotropinoma; U-SPH: unusual silent plurihormonal tumour; SPH- PIT1: silent plurihormonal.

Silent PitNET are pituitary tumours that immunohistochemically express specific pituitary hormones and cell specific transcription factors. So this type of tumours show ultrastructural features of a particular pituitary cell type [25]. Therefore, the identification of the silent functioning pituitary adenomas has mainly relied on their immunostaining pattern. Immunostaining in at least 5% of the tumour cell population has been considered relevant to a specific secretory pattern[26]. However, the percentage of the cell population with immunoreactivity varies widely among silent tumours, and frequently there is combined immunostaining of different pituitary hormones. Consequently, the gene expression of pituitary hormones should also be different in tumours of the same cell lineage, so their identification based on the dominant gene expression seems appropriate. Indeed, we did not find significant differences when we compared the IQR of gene expression between the functioning and non-functioning (silent) variants of the different subtypes of PitNET.

Identifying silent PitNET variants seems to be important[27]. SLT are very rare PitNET tumours, with only sporadic descriptions in the literature, mostly reporting their presentation together with Cushing adenomas[7,28]. Knowledge of silent lactotroph PitNET is also interesting because these could be treated with cabergoline. Thyrotroph PitNET are the least prevalent subtype, but their identification has clear therapeutic repercussions because they respond to treatment with SSA. As for STT, although they cannot benefit from the antisecretory effect of somatostatin analogues, the antiproliferative effect of this treatment can reduce or inhibit the progression of tumour remnants. The high number of STT detected molecularly, both in the present series and in the previous one, suggests that STT could be more prevalent than currently believed. In both series, molecular study was more sensitive than IHC in detecting the silent variants of both lactotroph and thyrotroph PitNET.

Of particular importance, the molecular study will allow us to typify the PitNET of our series according to the new 2017 WHO classification of anterior pituitary tumours[1]. The fourth edition of the World Health Organization (WHO) classification of endocrine tumours recommends classifying PitNET according to pituitary cell lineage. The purpose of this recommendation is to establish a practical classification system for clinician use, based on the immunochemistry of pituitary hormones and their pituitary-specific transcription factors. Moreover, in recognition of the classical PitNET subtypes, the new classification subdivides the PH tumours in Pit-1 positive PH tumours and tumours with unusual immunohistochemical combinations.

However, as with the previous classification of anterior pituitary tumours, the new classification relies again on immunohistochemistry, and it will probably generate the same between-centre variability in the interpretation of the results. Although in this study we did not analyse the pituitary-specific transcription factors, detecting the dominant expression of the pituitary-specific hormone genes allowed us to identify the two subtypes of PH tumours with more confidence than with the IHC study. The Pit1-PH tumour corresponds to the previously termed ‘silent-subtype 3 adenoma’, which behaves with unusual aggression[29]. These types of tumours can also behave as functioning PitNET. In a recent series of 31 silent subtype 3 pituitary tumours, 29% of the cases showed significant hormonal excess, including hyperthyroidism, acromegaly and hyperprolactinemia[30]. In the present series, 3 of 4 (75%) Pit1-PH tumours were functioning: they all presented acromegaly. Therefore, its correct identification has clinical consequences. Of course, studying the pituitary-specific transcription factors will permit a fuller recognition of the Pit1-PH tumours, but, in the meantime, the molecular study of pituitary-specific hormone genes could help in their identification.

The molecular identification of PH PitNET entails a risk from contamination of the tumour with normal pituitary tissue, so it is very important that an expert pathologist refers to the material on which the molecular studies should be carried out.

PitNET are one of the most common types of intracranial neoplasms[31]. Their prevalence in the general population is difficult to assess because the growing use and sensitivity of the image techniques is increasing the diagnosis of non-functioning PitNET. Clinically, prolactinomas are considered the most prevalent type of PitNET, followed by non-functioning PitNET and somatotroph, corticotroph and thyrotroph PitNET[32]. Nevertheless, from the pathology point of view, the prevalence of PitNET depends on the identification technique. As some subtypes of PitNET behave more aggressively than others, it is important to properly categorise the tumours and assess their prevalence. Pooling data from the two series, and excluding lactotroph PitNET, we found that the most prevalent subtype of molecularly identified PitNET was the gonadotroph (30.5%), followed by somatotroph (20.1%), corticotroph (18.5%), null cell (9.75%), plurihormonal (8.45%) and thyrotroph PitNET (6.45%). These numbers were different from those obtained by IHC, with a noticeable reduction in the percentage of null cell and plurihormonal PitNET in both series.

Strengths of our investigation include the reliability with which we reproduced the previous results published by Sanchez-Tejada *et al*. Like the previous study, a single researcher carried it out in a single centre. The series studied was specially selected on the basis of high-quality material provided by an expert pathologist and the availability of complete clinical, biochemical, radiological and IHC data for all patients.

On the other hand, the IHC took place in four participating centres, which makes an equitable comparison of both techniques difficult. The presence of gene expression does not guarantee the presence of protein expression. Therefore, despite the reliable information provided by the molecular study, its results should be complementary to the IHC study.

## Conclusions

This study reproduces, in an independent series, the results previously published by our group, enhancing the complementary role of the molecular study of the pituitary-specific hormone genes in the typification of PitNET subtypes. In addition, the two qRT-PCR techniques used are valid. Both techniques, TaqMan probes and SYBR Green primers, are suitable for the study of pituitary-specific hormone genes in PitNET. Although the molecular studies are unable to detect the densely and sparsely granulated variants of the PitNET, they help to reduce the number of null cell and plurihormonal tumours with an unequivocal clinical repercussion. Finally, we expect that the molecular study of the pituitary-specific transcription factors complements the IHC identification, ultimately improving the classification of PitNET subtypes.

## Supporting information

S1 Table Antibodies used in the immunohistochemical studies carried out in the pathology departments of the four participating hospitals. (DOC)

S2 Table Characteristics of the present series. (XLSX)

S3 Table Demographic and clinical characteristics of PitNET. (DOCX)

S4 Table Expression of the dominant specific genes of anterior pituitary hormones in the subtypes of PitNET. (DOCX)

S5 Table Median and IQR of GH gene expression in clinically functioning ST (with and without treatment with somatostatin analogues). (DOCX)

S6 Table Expression of the dominant pituitary-specific hormone genes in the subtypes of PitNET (functioning and silents). (DOCX)
