# Supplementary material for: Is it time to consider the expression of specific-pituitary hormone genes when typifying pituitary tumours?
Source: PLoS One. 2018 Jul 6;13(7):e0198877. doi: 10.1371/journal.pone.0198877 (PMC6034784; doi:10.1371/journal.pone.0198877)
Supplement: S1 Table — (DOC) [file pone.0198877.s001.doc]

Table S1. Antibodies used in the immunohistochemical studies carried out in the Pathology Departments of the four participating hospitals.

| **HORMONE** | **ANTIBODY USED** | **DILUTION** |
| --- | --- | --- |
| **Follicle-stimulating hormone** |  |  |
| Hospital 1 | Monoclonal Mouse Anti-Human FSH  Clone C10 – M3504 DAKO | 1:200 |
| Hospital 2 | FSH Menarini policlonal | 1:50 |
| Hospital 3 | Monoclonal Mouse Anti-Human FSH  Clone C10 – M3504 DAKO | 1:50 |
| Hospital 4 | Monoclonal Mouse Anti-Human FSH  Clone C10 – M3504 DAKO | 1:50 |
| **Luteinizing hormone** |  |  |
| Hospital 1 | Monoclonal Mouse Anti-Human LH  Clone C93 – M3502 DAKO | 1:300 |
| Hospital 2 | LH Menarini policlonal | 1:50 |
| Hospital 3 | Monoclonal Mouse Anti-Human LH  Clone C93 – M3502 DAKO | 1:50 |
| Hospital 4 | Monoclonal Mouse Anti-Human LH  Clone C93 – M3502 DAKO | 1:50 |
| **Alpha subunit** |  |  |
| Hospital 1 | 4E12. Novocastra | 1:100 |
| Hospitales 2-4 | Not done |  |
| **Prolactin** |  |  |
| Hospital 1 | Polyclonal Rabbit Anti-Human Prolactin  DAKO A0569 | 1:200 |
| Hospital 2 | Polyclonal Rabbit Anti-Human Prolactin  DAKO A0569 | 1:50 |
| Hospital 3 | Polyclonal Rabbit Anti-Human Prolactin  DAKO A0569 | 1:200 |
| Hospital 4 | Biomeda | 1:200 |
| **Adrenocorticotropic hormone** |  |  |
| Hospital 1 | Monoclonal Mouse Anti- ACTH  Clone 02A3 – M3501 DAKO | 1:500 |
| Hospital 2 | Monoclonal Mouse Anti- ACTH  Clone 02A3 – M3501 DAKO | 1:50 |
| Hospital 3 | Monoclonal Mouse Anti- ACTH  Clone 02A3 – M3501 DAKO | 1:100 |
| Hospital 4 | Monoclonal Mouse Anti- ACTH  Clone 02A3 – M3501 DAKO | 1:100 |
| **Growth hormone** |  |  |
| Hospital 1 | Polyclonal, Novocastra | 1:100 |
| Hospital 2 | HGH Dako polyclonal | 1:50 |
| Hospital 3 | HGH Dako polyclonal | 1:50 |
| Hospital 4 | HGH Dako polyclonal | 1:300 |
| **Thyroid stimulating hormone** |  |  |
| Hospital 1 | Monoclonal Mouse Anti-Human TSH  Clone 0042 – M3503 DAKO | 1:300 |
| Hospital 2 | Monoclonal Mouse Anti-Human TSH  Clone 0042 – M3503 DAKO | 1:50 |
| Hospital 3 | Monoclonal Mouse Anti-Human TSH  Clone 0042 – M3503 DAKO | 1:50 |
| Hospital 4 | Biomeda | 1:100 |
