# Supplementary material for: Is it time to consider the expression of specific-pituitary hormone genes when typifying pituitary tumours?
Source: PLoS One. 2018 Jul 6;13(7):e0198877. doi: 10.1371/journal.pone.0198877 (PMC6034784; doi:10.1371/journal.pone.0198877)
Supplement: S3 Table — (DOCX) [file pone.0198877.s003.docx]

Table S3. Demographic and clinical characteristics of PitNET.

| **PitNET** | **Total**  **% (n)** | **Female**  **% (n)** | **Age (years)**  **mean±SD** | **Macroadenoma**  **% (n)** |
| --- | --- | --- | --- | --- |
| **Functioning tumours** | **42.8% (48)** | **58.3% (28)** | **45.2±14.6** | **81.3% (39)** |
| FCT | 20.8% (10) | 90.0% (9) | 45.0±10.9 | 50.0% (5) |
| FST | 56.2% (27) | 59.3% (16) | 45.7±13.6 | 88.9% (24) |
| FLT | 16.7% (8) | 37.5% (3) | 44.5±20.8 | 87.5% (7) |
| FTT | 6.2% (3) | 0.0% (0) | 43.0±22.6 | 100.0% (3) |
| **Non Functioning tumours** | **57.2% (64)** | **35.9% (23)** | **56.1±15.6** | **98.4% (63)** |
| **Total** | **100.0% (112)** | **45.5% (51)** | **51.4±16** | **91.1% (102)** |
