# Supplementary material for: Is it time to consider the expression of specific-pituitary hormone genes when typifying pituitary tumours?
Source: PLoS One. 2018 Jul 6;13(7):e0198877. doi: 10.1371/journal.pone.0198877 (PMC6034784; doi:10.1371/journal.pone.0198877)
Supplement: S4 Table — (DOCX) [file pone.0198877.s004.docx]

| **PitNET Subtypes** | ***GH*** | ***FSH*** | ***LH*** | ***TSH*** | ***PRL*** | ***POMC*** | ***AVPR1B*** | ***CRHR1*** |
| --- | --- | --- | --- | --- | --- | --- | --- | --- |
| **ST (n=12)** | **1.225 (0.305-3.06)** | 0.002 (0.000-0.019) | 0.001 (0.000-0.006) | 0.071 (0.008-0.153) | 0.091 (0.028-0.176) | 0.015 (0.001-0.089) | 0.003 (0.001-0.029) | 0.031 (0.002-0.237) |
| **ST MIXED (n=7)** | **1.777 (0.719-35.749)** | 0.002 (0.001-0.019) | 0.003 (0.000-0.016) | 0.039 (0.009-0.085) | **1.632 (0.266-5.225)** | 0.003 (0.001-0.19) | 0.004 (0.000-0.08) | 0.056 (0.001-0.302) |
| **CT (n=13)** | 0.000 (0.000-0.000) | 0.000 (0.000-0.002) | 0.000 (0.000-0.000) | 0.002 (0.000-0.003) | 0.001 (0.000-0.016) | **1.067 (0.115-12.668)** | **3.296 (0.557-5.992)** | **3.566 (0.226-29.631)** |
| **LT (n=10)** | 0.001 (0.001-0.026) | 0.001 (0.000-0.008) | 0.000 (0.000-0.002) | 0.029 (0.003-0.126) | **4.355 (2.354-17.939)** | 0.012 (0.000-0.155) | 0.024 (0.000-0.364) | 0.216 (0.002-0.267) |
| **LT STEM (n=1)** | **0.324 (0.324-0.324)** | 0.077 (0.077-0.077) | 0.055 (0.055-0.055) | 0.006 (0.006-0.006) | **5.091 (5.091-5.091)** | 0.012 (0.012-0.012) | 0.018 (0.018-0.018) | 0.054 (0.054-0.054) |
| **TT (n=9)** | 0.019 (0.001-0.046) | 0.033 (0.000-0.202) | 0.001 (0.000-0.013) | **3.678 (2.148-11.703)** | 0.002 (0.000-0.115) | 0.002 (0.000-0.019) | 0.016 (0.000-0.395) | 0.046 (0.004-0.29) |
| **GT (n=31)** | 0.000 (0.000-0.000) | **1.339 (0.365-3.769)** | **0.004 (0.001-0.024)** | 0.012 (0.004-0.044) | 0.000 (0.000-0.002) | 0.000 (0.000-0.001) | 0.002 (0.000-0.005) | 0.051 (0.016-0.141) |
| **NC (n=14)** | 0.000 (0.000-0.000) | 0.029 (0.001-0.067) | 0.000 (0.000-0.005) | 0.004 (0.001-0.012) | 0.000 (0.000-0.000) | 0.000 (0.000-0.000) | 0.001 (0.000-0.011) | 0.024 (0.016-0.127) |
| **U-PH (n=11)** | **0.123 (0.002-0.852)** | **1.048 (0.109-3.503)** | **0.019 (0.001-1.084)** | **2.266 (0.032-6.076)** | **0.858 (0.244-5.854)** | 0.012 (0.000-1.262) | 0.005 (0.000-0.705) | 1.641 (0.088-3.423) |
| **PH PIT1 (n=4)** | **0.076 (0.031-0.179)** | 0.000 (0.000-0.037) | 0.000 (0.000-0.046) | **1.481 (0.883-2.838)** | **1.138 (0.149-2.266)** | 0.000 (0.000-0.149) | 0.001 (0.000-0.123) | 0.,12 (0.000-1.245) |

Table S4. Expression of the dominant specific genes of anterior pituitary hormones in the subtypes of PitNET*.

*The variable was expressed as median (p25-p75).
