# Supplementary material for: Is it time to consider the expression of specific-pituitary hormone genes when typifying pituitary tumours?
Source: PLoS One. 2018 Jul 6;13(7):e0198877. doi: 10.1371/journal.pone.0198877 (PMC6034784; doi:10.1371/journal.pone.0198877)
Supplement: S5 Table — (DOCX) [file pone.0198877.s005.docx]

Table S5. Median and IQR of GH gene expression in clinically functioning ST (with and without treatment with somatostatin analogues).

| **Clinically FST** | ***GH*** (median (p25-p75)) | ***p-value*** |
| --- | --- | --- |
| Global series | 0.719 (0.174-2.329) |  |
| With treatment | 0.629 (0.176-1.684) | *0.446** |
| Without treatment | 1.169 (0.197-3.304) |  |

*Mann Whitney test.
