# Supplementary material for: Is it time to consider the expression of specific-pituitary hormone genes when typifying pituitary tumours?
Source: PLoS One. 2018 Jul 6;13(7):e0198877. doi: 10.1371/journal.pone.0198877 (PMC6034784; doi:10.1371/journal.pone.0198877)
Supplement: S6 Table — (DOCX) [file pone.0198877.s006.docx]

Table S6. Expression of the dominant pituitary-specific hormone genes in the subtypes of PitNET (functioning and silents)*.

| **PitNET** | ***GH*** | ***FSH*** | ***LH*** | ***TSH*** | ***PRL*** | ***POMC*** | ***AVPR1B*** | ***CRHR1*** |
| --- | --- | --- | --- | --- | --- | --- | --- | --- |
| **FST (n=12)** | **1.225 (0.305-3.06)** | 0.002 (0.000-0.019) | 0.001 (0.000-0.006) | 0.071 (0.008-0.153) | 0.091 (0.028-0.176) | 0.015 (0.001-0.089) | 0.003 (0.001-0.029) | 0.031 (0.002-0.237) |
| **FST MIXED (n=7)** | **1.777 (0.719-35.749)** | 0.002 (0.001-0.019) | 0.003 (0.000-0.016) | 0.039 (0.009-0.085) | **1.632 (0.266-5.225)** | 0.003 (0.001-0.19) | 0.004 (0.000-0.08) | 0.056 (0.001-0.302) |
| **FCT (n=7)** | 0.000 (0.000-0.000) | 0.001 (0.000-0.002) | 0.000 (0.000-0.001) | 0.002 (0.000-0.009) | 0.001 (0.000-0.002) | **11.722 (1.067-21.887)** | **3.296-0.826-24.512)** | **19.928 (1.192-42.651)** |
| **SCT (n=6)** | 0.000 (0.000-0.000) | 0.000 (0.000-0.000) | 0.000 (0.000-0.000) | 0.000 (0.000-0.004) | 0.002 (0.000-0.012) | **0.121 (0.001-3.357)** | **3.005 (0.001-5.446)** | **0.226 (0.007-14.358)** |
| **FLT (n=4)** | 0.000 (0.000-0.09) | 0.001 (0.000-0.003) | 0.000 (0.000-0.000) | 0.009 (0.001-0.114) | **10.296 (4.119-22.289)** | 0.002 (0.000-0.016) | 0.007 (0.000-0.024) | 0.007 (0.000-0.172) |
| **SLT (n=6)** | 0.011 (0.001-0.08) | 0.001 (0.000-0.039) | 0.000 (0.000-0.012) | 0.041 (0.003-0.144) | **2.97 (0.859-36.752)** | 0.120 (0.000-0.387) | 0.170 (0.001-0.524) | 0.219 (0.161-0.578) |
| **FLT STEM (n=1)** | **0.324 (0.324-0.324)** | 0.077 (0.077-0.077) | 0.055 (0.055-0.055) | 0.006 (0.006-0.006) | **5.091 (5.091-5.091)** | 0.012 (0.012-0.012) | 0.018 (0.018-0.018) | 0.054 (0.054-0.054) |
| **FTT (n=3)** | 0.019 (0.005-0.052) | 0.103 (0.002-0.205) | 0.000 (0.000-0.001) | **17.343 (3.678-19.886)** | 0.000 (0.000-0.024) | 0.001 (0.000-0.002) | 0.001 (0.000-0.003) | 0.004 (0.002-0.013) |
| **STT (n=6)** | 0.011 (0.001-0.049) | 0.033 (0.000-0.247) | 0.005 (0.000-0.018) | **2.298 (1.938-5.504)** | 0.040 (0.001-0.171) | 0.010 (0.001-0.170) | 0.034 (0.015-1-213) | 0.239 (0.081-0.52) |
| **U-FPH (n=3)** | **1.591 (0.261-2.156)** | **0.109 (0.006-0.257)** | **0.351 (0.001-1.084)** | **0.007 (0.004-6.076)** | **0.382 (0.048-5.854)** | **1.262 (0.351-6.468)** | **0.705 (0.287-0.926)** | **3.423 (2.402-4.566)** |
| **U-SPH (n=8)** | **0.008 (0.001-0.212)** | **1.946 (0.533-3.605)** | **0.011 (0.000-1.257)** | **2.883 (0.148-9.828)** | **1.667 (0.354-6.942)** | **0.005 (0.000-0.147)** | **0.002 (0.000-0.018)** | **0.132 (0.052-2.101)** |
| **FPH PIT1 (n=3)** | **0.109 (0.044-0.203)** | 0.000 (0.000-0.001) | 0.000 (0.000-0.000) | **1.203 (0.776-3.198)** | **0.355 (0.081-1.921)** | 0.000 (0.000-0.001) | 0.001 (0.000-0.001) | 0.002 (0.000-1.58) |
| **SPH PIT1 (n=1)** | **0.027 (0.027-0.027)** | 0.049 (0.049-0.049) | 0.062 (0.062-0.062) | **1.76 (1.76-1.76)** | **2.381 (2.381-2.381)** | 0.198 (0.198-0.198) | 0.164 (0.164-0.164) | 0.239 (0.239-0.239) |
| **GT (n=31)** | 0.000 (0.000-0.000) | **1.339 (0.365-3.769)** | **0.004 (0.001-0.024)** | 0.012 (0.004-0.044) | 0.000 (0.000-0.002) | 0.000 (0.000-0.001) | 0.002 (0.000-0.005) | 0.051 (0.016-0.141) |
| **NC (n=14)** | 0.000 (0.000-0.000) | 0.029 (0.001-0.067) | 0.000 (0.000-0.005) | 0.004 (0.001-0.012) | 0.000 (0.000-0.000) | 0.000 (0.000-0.000) | 0.001 (0.000-0.011) | 0.024 (0.016-0.127) |

*The variable was expressed as median (p25-p75).
